# Special Issue “Phyto-Functional Nanomaterials: Synthesis, Characterization and Application”

**DOI:** 10.3390/ijms27135878

**Published:** 2026-06-30

**Authors:** Nuno M. M. Moura, Carla I. M. Santos, Maria G. P. M. S. Neves

**Affiliations:** 1 REQUIMTE, Department of Chemistry, University of Aveiro, Campus Universitário de Santiago, 3810-193 Aveiro, Portugal; 2 Centro de Química Estrutural, Institute of Molecular Sciences, and Departamento de Engenharia Química, Instituto Superior Técnico, Universidade de Lisboa, 1049-001 Lisboa, Portugal; carla.santos@tecnico.ulisboa.pt

## 1. Phyto-Functional Nanomaterials for Sustainable Applications

While the transition toward sustainable technologies in energy, environmental protection, and biomedicine has brought nanomaterials to the center of contemporary materials research [1,2,3], the success of nanotechnology has exposed an uncomfortable paradox: many of the nanostructures that promise cleaner energy, safer water, and more effective therapies are still produced through synthetic routes that depend on hazardous reducing agents, toxic solvents, energy-intensive procedures, and non-renewable feedstocks [4,5]. Adapting the performance of modern nanomaterials to the principles of green chemistry has therefore become one of the most pressing objectives in molecular nanoscience [4,6,7].

Within this context, phyto-functional nanomaterials provide a credible approach for addressing this issue. By exploiting plant extracts, algal biomass, agro-industrial residues, and other biogenic sources, researchers have shown that it is possible to obtain nanostructures with controlled size, shape, and surface chemistry using water as the main solvent and naturally occurring phytochemicals as reducing and stabilizing agents [8,9,10]. Polyphenols, flavonoids, terpenoids, alkaloids, polysaccharides, proteins, and mycosporine-like amino acids, among other secondary metabolites, fulfill several roles simultaneously: they reduce metal precursors, cap and stabilize the resulting nanoparticles, modulate dispersibility and morphology, and frequently contribute to the final material through their own intrinsic bioactivity (e.g., antioxidant, antimicrobial, photoprotective, or anticancer activities) [8,10]. The outcome is a class of nanostructures that is not only greener to produce, but is often inherently multifunctional [9,11]. Although several reviews have already surveyed the principles of green nanoparticle synthesis, the present collection expands upon this body of work along three complementary axes: it brings together biogenic synthesis, bio-based processing, and device-level integration; it places experimental advances alongside mechanistic and theoretical studies; and it follows the materials from synthesis to performance-oriented applications in energy, environment, and biomedicine.

**Figure 1:**
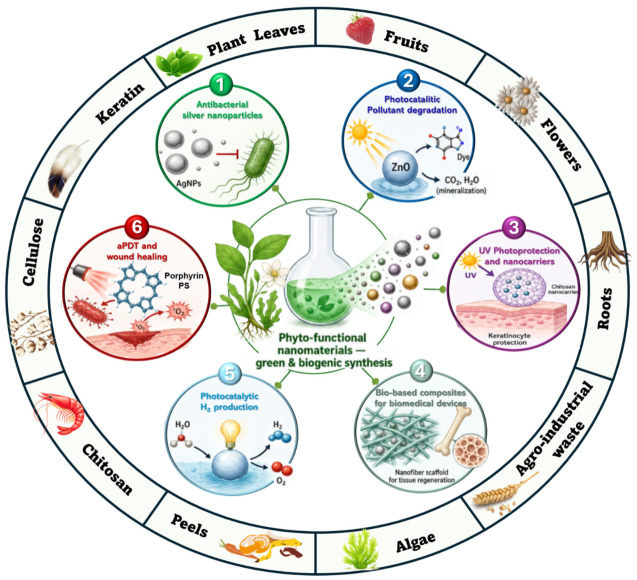
Spectrum of applications discussed in this Special Issue, ranging from photocatalytic water splitting and pollutant degradation to antimicrobial photodynamic therapy, wound management, photoprotection of human skin, and biomedical composites.

This Special Issue, “Phyto-Functional Nanomaterials: Synthesis, Characterization and Application”, was conceived in 2023 as a collection of original research and authoritative reviews reflecting the dimension and maturity of this rapidly evolving field. The nine contributions collected here—seven original research articles and two comprehensive reviews—cover synthetic strategies, characterization approaches, and applications ranging from photocatalytic water splitting and pollutant degradation to antimicrobial photodynamic therapy, wound management, photoprotection of human skin, and biomedical composites for tissue engineering (Figure 1).

## 2. Green and Biogenic Synthesis of Metal and Metal-Oxide Nanoparticles

Three contributions included in this Special Issue highlight the growing maturity of plant-mediated approaches for the synthesis of metal and metal-oxide nanoparticles, showing how biogenic strategies have moved beyond proof of concept toward more controlled and application-driven methodologies.

Al-Audah et al. (Contribution 1) explore the aqueous fruit extract of *Malva parviflora* as a biological resource for the green synthesis of silver nanoparticles (AgNPs). This work draws attention to the still largely untapped potential of regional plant biodiversity, where distinct phytochemical compositions can influence nanoparticle formation, stability, and functionality. The biosynthesized AgNPs exhibited a characteristic surface plasmon resonance peak and anisotropic morphologies, including rod- and needle-like structures. Phytochemical analysis identified hydroxyl, carbonyl, amide, and phenyl groups acting in both reduction and stabilization, and the particles displayed a negative surface charge consistent with colloidal stability. The AgNPs showed pronounced antibacterial activity at relatively low bactericidal concentrations against multidrug-resistant strains, including methicillin-resistant *Staphylococcus aureus* (MRSA) and extended-spectrum β-lactamase-producing *Escherichia coli* (ESBL), despite a comparatively large particle size, suggesting the possible role of morphology and phytochemical capping in modulating biological interactions. Antioxidant and anti-inflammatory activities, together with controlled cytotoxicity profiles, further support the multifunctional character of the material. Further work is warranted to clarify the mechanisms of action, improve control over particle size distribution, and validate the findings in relevant in vivo models.

Andziukevičiūtė-Jankūnienė et al. (Contribution 2) combined biosynthesized silver nanoparticles (~20.5 nm) produced with *Echinacea purpurea* L. extract with electrospun mats of wool-derived keratin and poly(ethylene oxide). The synthesized bioAgNPs exhibited a concentration-dependent inhibition of both Gram-positive and Gram-negative bacteria, while the incorporation of either the plant extract or the nanoparticles into the spinning solution modulated its rheology, shifting fiber diameters toward the 50–100 nm range. The authors show that polyphenols from the *Echinacea* extract play a dual role, acting both as electron donors during Ag^+^ bioreduction and as stabilizers of the resulting nanoparticles, exemplifying the multifunctionality of phytochemicals highlighted in the introduction. The resulting antibacterial textile-like materials, built entirely from renewable components, show how green nanosynthesis can be coupled with electrospinning to deliver fully bio-based composite devices for wound dressings and biomedical textiles.

Purushotham et al. (Contribution 3) extend the green-synthesis paradigm from metals to semiconducting oxides. Using *Pavonia zeylanica* leaf extract, they obtained zinc oxide nanoparticles (~19.6 nm, bandgap 3.04 eV) with a well-defined wurtzite structure, and FT-IR analysis confirmed the contribution of phytoconstituents to both synthesis and stabilization. Under solar irradiation at neutral pH and room temperature, the Pz-ZnO-NPs achieved 89.3% photodegradation of methylene blue within two hours, following pseudo-first-order kinetics. The authors go beyond a simple demonstration of decolorization: they assess recyclability across five consecutive cycles, where 82.8% degradation efficiency was retained while preserving the wurtzite structure, and quantify the actual extent of mineralization through chemical oxygen demand (COD) and total organic carbon (TOC) measurements. The results provide evidence that the dye suffered degradation to non-toxic products rather than being merely adsorbed. This kind of quantitative, application-oriented characterization, combined with the use of natural solar light rather than artificial UV sources, moves us closer towards the use of biogenic photocatalysts in real-world wastewater treatment.

## 3. Photocatalysis and Fundamental Mechanistic Insights

While most of the included contributions are experimentally driven, theoretical and mechanistic understanding remains essential for the rational design of efficient photocatalysts, including the biogenic and bio-hybrid systems at the center of this Special Issue. Pang et al. (Contribution 4) address this by applying Landau–Zener trajectory surface hopping molecular dynamics at the ADC(2) level to a heptazine–(H_2_O)_4_ cluster, a model system relevant to carbon-nitride photocatalysts for hydrogen evolution. Their simulations confirm the electron-driven proton transfer (EDPT) mechanism from water to the heptazine chromophore, yielding a heptazinyl radical and a hydroxyl biradical, with a calculated quantum yield of 6.5%, which is only modestly below the 9% obtained for the single-water complex, suggesting that enlarging the water cluster does not substantially enhance hydrogen transfer efficiency. The authors further identify two distinct decay regimes (fast, tens of femtoseconds; slow, several hundred femtoseconds), with the crossover between N–H and O–H bond lengths emerging as the critical marker of the population-hopping event. These molecular-level insights offer theoretical guidance that complement ongoing experimental progress in heptazine and polymeric carbon nitride photocatalysts, increasingly explored in combination with bio-derived components.

## 4. Phyto-Derived and Bio-Based Nanoformulations for Biomedical Applications

A second group of contributions focuses on nanoformulations that explicitly capitalize on the intrinsic bioactivity of natural compounds, rather than using phytochemicals merely as synthetic auxiliaries.

Vásquez and co-workers (Contribution 5) report on chitosan nanoformulations (CSNFs) loaded with mycosporine-like amino acid (MAA)-rich aqueous extracts from the Chilean red alga *Mazzaella laminarioides*, locally known as *luga cuchara*. MAAs are increasingly recognized as natural alternatives to synthetic UV filters, and the authors position them as eco-friendly substitutes for chemical filters such as oxybenzone, which have raised growing concerns related to human and marine ecosystem toxicity. Using a fully aqueous, solvent-free extraction protocol, an MAA-rich extract was encapsulated into chitosan nanoparticles with high encapsulation efficiency. In HaCaT keratinocytes, pre-treatment with CSNFs conferred complete protection against low-to-moderate UVA doses and retained substantial protection efficacy under a lethal dose equivalent to more than twice the UVA exposure of midday summer sun in southern Europe. The formulations were also able to restore keratinocyte viability in post-treatment assays, operating at MAA concentrations well below those used in conventional sunscreens. Gene-expression analysis identified the activation of compensatory cytoprotective pathways, adding mechanistic depth beyond the phenomenological photoprotection. This work demonstrates how polysaccharide-based nanocarriers can stabilize and deliver fragile marine bioactives, and positions marine biomass as a credible source of sustainable photoprotective agents.

Maternia-Dudzik et al. (Contribution 6) investigate nanocellulose-modified poly(3-hydroxybutyrate) (P3HB) composites, combining a bacterially produced biopolyester (synthesized by *Ralstonia eutropha* H16) with plant-derived nanocrystalline cellulose, bringing together two fully renewable, biogenic feedstocks of distinct biological origin. P3HB is a biodegradable polyester whose mechanical brittleness and narrow processing window have long limited its biomedical applications. The authors show that very low filler loadings (0.5–1 wt%) are sufficient to substantially improve the properties of the matrix, with the optimal composition (P3HB-1) achieving the widest processing window and the most plastic character while maintaining tensile strength close to that of the neat polymer. DSC, TGA, XRD, SEM, and mechanical testing together reveal a non-trivial structure–property relationship: nanocellulose reduces crystallinity in the amorphous-dominated regime but acts as a nucleating agent at higher loadings, with corresponding effects on stiffness and deformability. Importantly, the work also addresses an aspect too often overlooked in nanocomposite studies: biological safety. Immunosafety assessments aligned with FDA and EMA guidance confirmed either no or very low endotoxin contamination, and biological tests for implant applications identified the 0.5 wt% formulation as the most promising. This study shows how two renewable, fully bio-based components—one of bacterial and another of plant origin—can be combined to produce nanocomposites tailored to the demanding requirements of biomedical devices, and illustrates that compositional fine-tuning at the sub-percent level can be more decisive than the choice of components themselves.

## 5. Antimicrobial Photodynamic Strategies

Antimicrobial photodynamic therapy (aPDT) has emerged as one of the most promising light-activated alternatives to conventional antibiotics, particularly in the context of the global crisis of antimicrobial resistance [12,13,14,15,16]. Moura and co-workers (Contribution 7) describe efficient strategies based on two β-modified monocharged porphyrin–imidazolium derivatives, incorporated either into polyvinylpyrrolidone (PVP) formulations or immobilized onto graphitic carbon nitride (GCN) supports. The combination of cationic charges and imidazolium functionalities enhanced the affinity of the photosensitizers (PS) for bacterial membranes, while retaining the high photostability and singlet-oxygen generation efficiency required for an effective photodynamic action. Both formulations achieved 99.9999% photoinactivation of methicillin-resistant *Staphylococcus aureus* (MRSA): the PVP-based formulation required only 10 min of white-light irradiation at 5.0 µM PS, while the GCN-supported hybrid reached comparable efficacy at 25 µM after 20 min, with the added advantage of catalyst recovery after treatment and avoidance of organic solvents. This work shows how rational molecular design, coupled with judicious choice of supporting matrix, can yield effective antimicrobial agents that act through mechanisms less prone to classical resistance pathways, with prospective applications ranging from blood and plasma disinfection to skin infection treatment and environmental decontamination. The use of graphitic carbon nitride as a porphyrin support also connects to the mechanistic study of Pang et al. (Contribution 4), highlighting the dual relevance of GCN-based materials as both photocatalysts for water splitting and scaffolds for antimicrobial photodynamic action. Graphitic carbon nitride itself can notably be obtained from bio-derived precursors such as urea, chitosan, sucrose, or nitrogen-rich agricultural waste [17,18,19,20], reinforcing the sustainability credentials of the GCN-supported hybrid.

## 6. Reviews Bridging Biopolymers, Photosensitizers, and Clinical Needs

Two review articles consolidate and contextualize recent progress in the field, with a deliberate focus on natural biopolymers as renewable scaffolds for advanced nanocomposites.

Bielska and Miłowska (Contribution 8) provide a comprehensive overview of chitosan-based nanocomposite systems for wound management, with particular attention to the synergy between chitosan nanostructures and natural polyphenols. Chronic wounds, particularly those associated with diabetes, remain a major clinical and economic challenge due to prolonged or incomplete healing, elevated infection rates, and the ensuing risk of lower-limb amputation. This review examines how the encapsulation of polyphenols such as epigallocatechin gallate (EGCG), quercetin, and curcumin within chitosan nanostructures simultaneously overcomes two well-known limitations: the poor aqueous solubility and instability of polyphenols, and the relatively modest standalone biological performance of chitosan. The resulting multifunctional systems combine the controlled and localized release of bioactives with the intrinsic biocompatibility, biodegradability, and antimicrobial activity of the polymeric matrix, and can be further enhanced through the incorporation of silver nanoparticles, gelatin, or plant-derived extracts to deliver synergistic antimicrobial protection and tissue regeneration. The authors carefully map the biological mechanisms involved, such as the promotion of angiogenesis, fibroblast proliferation, epithelial regeneration, and the modulation of oxidative stress and inflammation, while acknowledging that most of the available evidence is still preclinical and that translational success will require deeper mechanistic understanding and rigorous quantitative evaluation. The review offers both a practical roadmap for the rational design of chitosan–polyphenol–metal nanoparticle hybrids and a balanced perspective on what remains to be done before such systems can reach clinical practice.

Monteiro et al. (Contribution 9) review the grafting of porphyrin PS onto cellulose and cellulose-derivative supports, illustrating how one of the most abundant natural biopolymers can be transformed into functional, antimicrobial, and biomedically relevant materials. The authors systematically cover the three main immobilization strategies—adsorption, entrapment, and covalent linkage—and pay particular attention to the synthetic routes required to functionalize both partners: chemical modification of cellulose to introduce reactive handles, and structural tailoring of porphyrins with linker groups that enable robust grafting. The resulting photoactive hybrids combine the intrinsic advantages of cellulose-based matrices (high surface area, improved mechanical strength, barrier properties, biocompatibility, and renewable origin) with the visible-light-triggered reactive oxygen species chemistry of porphyrinoid PS, opening practical routes to self-disinfecting surfaces, antimicrobial textiles, photoactive wound dressings for diabetic feet, pressure ulcers, and burn wounds, and antimicrobial coatings for gloves, catheters, and food-packaging materials. A brief but valuable section also extends the discussion to cellulose-supported PS for cancer photodynamic therapy, broadening the scope beyond antimicrobial action. Importantly, the review highlights an often-overlooked sustainability feature of these systems—the possibility of recovering and reusing the PS–cellulose hybrid after treatment—and identifies clear directions for further work, including the rational tuning of PS amphiphilicity and membrane-targeting properties, as well as the development of site-directed delivery strategies.

Together with the review by Bielska and Miłowska (Contribution 8) on chitosan-based wound management systems, this contribution underscores the central role of natural biopolymers as renewable scaffolds for the next generation of antimicrobial and regenerative materials and complements the experimental study of Moura et al. (Contribution 7) by mapping the broader landscape into which such photoactive molecules can be integrated.

## 7. Concluding Remarks

Taken as a whole, the nine contributions in this Special Issue illustrate three converging trends in the field. First, the substitution of hazardous reagents by plant extracts, algal biomass, and other biogenic sources is no longer a niche curiosity but is rather a reproducible strategy capable of yielding nanomaterials with well-defined and tunable properties. Second, the resulting phyto-functional nanostructures are inherently multifunctional: the same material may simultaneously act as a photocatalyst, an antimicrobial agent, a photoprotective carrier, and a biocompatible scaffold, blurring the traditional boundaries between energy, environmental, and biomedical applications. Third, sustained progress in this field increasingly depends on the integration of experimental synthesis and characterization with mechanistic and theoretical studies, such that empirical advances are accompanied by a deeper molecular-level understanding of structure–property–activity relationships.

Several challenges nevertheless remain before phyto-functional nanomaterials can realize their full translational potential. The batch-to-batch variability of plant and algal extracts continues to complicate reproducibility and regulatory acceptance; the scale-up of biogenic syntheses beyond the laboratory bench is still largely unexplored; and rigorous life-cycle assessments are needed to confirm that “green” routes deliver genuine environmental benefits over the full production chain, from feedstock cultivation to end-of-life disposal. Addressing these issues will require closer collaboration between chemists, biologists, engineers, and policymakers, and will benefit from the integration of data-driven and computational tools to standardize, predict, and optimize phyto-functional nanomaterial properties.

The contributions also reflect the international scope of the field, bringing together research groups from Europe, the Americas, the Middle East, and Asia.
